# A methodological framework for AI-assisted diagnosis of active aortitis using radiomic analysis of FDG PET–CT images: Initial analysis

**DOI:** 10.1007/s12350-022-02927-4

**Published:** 2022-03-23

**Authors:** Lisa Duff, Andrew F. Scarsbrook, Sarah L. Mackie, Russell Frood, Marc Bailey, Ann W. Morgan, Charalampos Tsoumpas

**Affiliations:** 1 Leeds Institute of Cardiovascular and Metabolic Medicine, University of Leeds, 8.49b Worsley Building, Clarendon Way, Leeds, LS2 9JT UK; 2 Institute of Medical and Biological Engineering, University of Leeds, Leeds, UK; 3 Leeds Institute of Medical Research - St James’s, University of Leeds, Leeds, UK; 4 Department of Radiology, St. James University Hospital, Leeds, UK; 5 Leeds Institute of Rheumatic and Musculoskeletal Medicine, University of Leeds, Leeds, UK; 6 Leeds Teaching Hospitals NHS Trust, Biomedical Research Centre, NIHR Leeds, Leeds, UK; 7 The Leeds Vascular Institute, Leeds General Infirmary, Leeds, UK; 8 Icahn School of Medicine at Mount Sinai, Biomedical Engineering and Imaging Institute, New York, USA; 9 Department of Nuclear Medicine and Molecular Imaging, University Medical Center of Groningen, University of Groningen, 9700 RB Groningen, Netherlands

**Keywords:** Large-vessel vasculitis, FDG PET/CT, Radiomic feature analysis, Diagnosis, Giant cell arteritis

## Abstract

**Background:**

The aim of this study was to explore the feasibility of assisted diagnosis of active (peri-)aortitis using radiomic imaging biomarkers derived from [^18^F]-Fluorodeoxyglucose Positron Emission Tomography–Computed Tomography (FDG PET–CT) images.

**Methods:**

The aorta was manually segmented on FDG PET–CT in 50 patients with aortitis and 25 controls. Radiomic features (RF) (*n* = 107), including SUV (Standardized Uptake Value) metrics, were extracted from the segmented data and harmonized using the ComBat technique. Individual RFs and groups of RFs (i.e., signatures) were used as input in Machine Learning classifiers. The diagnostic utility of these classifiers was evaluated with area under the receiver operating characteristic curve (AUC) and accuracy using the clinical diagnosis as the ground truth.

**Results:**

Several RFs had high accuracy, 84% to 86%, and AUC scores 0.83 to 0.97 when used individually. Radiomic signatures performed similarly, AUC 0.80 to 1.00.

**Conclusion:**

A methodological framework for a radiomic-based approach to support diagnosis of aortitis was outlined. Selected RFs, individually or in combination, showed similar performance to the current standard of qualitative assessment in terms of AUC for identifying active aortitis. This framework could support development of a clinical decision-making tool for a more objective and standardized assessment of aortitis.

**Supplementary Information:**

The online version contains supplementary material available at 10.1007/s12350-022-02927-4.

## Introduction

Aortitis is an inflammatory syndrome affecting the aorta and its major branches and can be caused by various diseases, including giant cell arteritis (GCA), Takayasu arteritis, isolated aortitis, and peri-aortic inflammation (inflammation, retroperitoneal fibrosis, IgG4-related disease).^1,2^ Most cases of aortitis are treated initially with glucocorticoids. Glucocorticoid therapy carries a risk of toxicity^3–6^ and it is therefore important that treatment is based on an accurate diagnosis. Diagnosis of active aortitis can be challenging, particularly for patients who have started treatment or have atherosclerosis, as symptoms and blood tests are non-specific.

**Figure 1:**
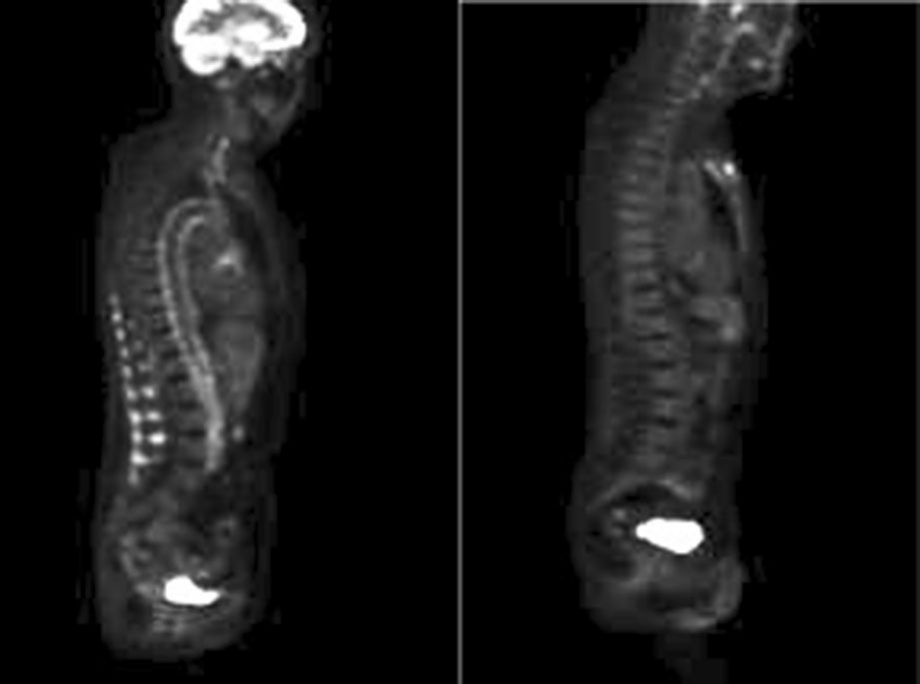
(Left) Sagittal FDG PET image of a patient with active aortitis. (Right) Sagittal FDG PET image of a control patient

[^18^F]-Fluorodeoxyglucose Positron Emission Tomography–Computed Tomography (FDG PET–CT) identifies areas of increased glycolytic activity in the inflamed vessel wall (Figure 1). FDG PET–CT is often used to assess patients with suspected aortitis due to large-vessel vasculitis (LVV):^7–9^ imaging guidelines advocate grading of FDG activity within the wall of major arteries.^10^. This qualitative grading is based on visual assessment by imaging specialists but this subjective evaluation can be inconsistent.^10–13^ Semi-quantitative parameters, objectively derived, are also frequently assessed, most commonly the standardized uptake value (SUV) mean (SUV_mean_) or maximum (SUV_max_). However, SUV measurements are influenced by many factors, including image noise, glucose concentration in plasma, and body habitus.^10^ Radiomics is a pattern recognition technique involving extraction of information, from medical images, referred to as radiomic features (RF) which may help better understand and stratify disease.^13–15^ These features range from simple, e.g., SUV metrics, to more complex descriptors of the shape and spatial relationships between individual voxels. While the biological correlate of individual RFs is yet to be fully elucidated, there is a renewed vigor for biological validation to become standard practice which could allow more definitive understanding and it is possible that a radiomics approach could be more discriminatory than conventional methods, e.g., for distinguishing inflammation from atherosclerosis and have a role in clinical decision-making^16^

The purpose of this study is to evaluate the feasibility and explore the potential utility of RFs extracted from FDG PET–CT for improving the accuracy of detecting active aortitis. The methodological framework combined RFs and machine learning (ML) classifiers to develop a prototype and rigorous semi-automated analysis tool.^17^

## Methods

Adherence to TRIPOD criteria (transparent reporting of a multivariable prediction model for individual prognosis or diagnosis)^18^ is detailed in Online Resource 1.

### Ethical Approval

The institutional research data access committee confirmed that formal ethics committee approval was not required for this study which was considered to represent evaluation of an established clinical service. Routinely collected patient meta-data were extracted by the clinical direct care team and rendered pseudo-anonymous for the purposes of analysis within this study. The institutional clinical governance team confirmed that this was also exempt from formal research ethics committee approval.

Prospective written consent was obtained from all patients at the time of imaging for use of their anonymized FDG PET–CT imaging data in research and service development projects. All patient data were prospectively entered into a departmental database used for retrospective identification and audit.

### Patient Selection

Patients with a systemic inflammatory response (pyrexia of unknown origin, high acute-phase response, weight loss) or suspected active aortitis undergoing FDG PET–CT were identified retrospectively from a single institution, Leeds Teaching Hospitals NHS Trust, between January 2011 and December 2019. The ground truth diagnoses for all patients and controls were confirmed by a consultant rheumatologist with 17 years’ experience of vasculitis (co-author AWM) based on clinical assessment, blood tests, biopsies, and qualitative assessment of FDG PET–CT scans by a dual-certified radiologist and nuclear medicine physician (co-author AFS) with more than 15 years’ experience of reporting FDG PET–CT. Exclusion criteria included synchronous metabolically active conditions obscuring or interfering with the aorta, such as malignancy. Patients with known LVV were excluded if they did not have imaging evidence of active aortitis. Control patients were excluded if they had activity in the aorta related to atherosclerosis. For LVV patients who had undergone multiple FDG PET scans, only the first scan that showed aortitis was selected. This study included a combination of newly diagnosed patients and patients with relapse. The imaging data for the selected aortitis patients (*n* = 50) and controls (*n* = 25) were extracted from the institutional PACS (Picture Archiving and Communication System) and pseudo-anonymized.

### Imaging Protocol

FDG PET–CT scans were acquired using a standard protocol: images were acquired from the upper thighs to the skull vertex.^10,19,20^ Imaging was acquired on three scanners during the study period, including a 64-slice Gemini TF64 scanner (Philips Healthcare, Best, the Netherlands; *n* = 29), a 64-slice Discovery 690 scanner (GE Healthcare, Chicago, IL, USA; *n* = 12), or a 64-slice Discovery 710 scanner (GE Healthcare, Chicago, IL, USA; *n* = 34). The images were reconstructed with iterative reconstruction algorithms and were corrected for attenuation, scatter, and randoms. Image reconstruction parameters for the different scanners are shown in Online Resource 2. Acquisition and reconstruction parameters were the same for all patients within each scanner.

### Segmentation

The entire aorta was manually segmented using 3D Slicer (Version 4.10.2, https: //www.slicer.org/) on the FDG PET–CT scan of each patient.^21,22^ Segmentation was conducted by a single observer (Author LD, Physics and Engineering researcher, limited experience) under supervision of co-author AFS. An initial batch (*n* = 15) of segmented volumes was validated against those performed by a clinical radiologist with 3 years’ of experience (acknowledged PA) to confirm inter-observer concordance. Dice Similarity Coefficients (DSC) were used for contour comparison. The PET images and segmented masks were then resampled to a 4-mm isotropic voxel size to ensure uniform sampling across the entire cohort. This voxel size was selected as it was the lowest resolution of the 3 scanners.

### Feature Extraction

Pyradiomics (Version 3.0.1, //www.radiomics.io/pyradiomics.html) was used to extract 102 RFs from the entire 3D volume of the segmented aorta in the PET images.^23^ Pyradiomics complies with the IBSI standards for most RFs and SUV metrics; any minor deviations are clearly described in their documentation (https://pyradiomics.readthedocs.io/en/latest/). All unfiltered features available through Pyradiomics were used. The SUV bin width was set to 0.075 in the Pyradiomics parameter input file. This bin width was selected by finding the max SUV value in the ROIs and dividing it by 64, a commonly used bin number in radiomics. No additional filters were used, and all other parameters were left as default. Five SUV features not included in Pyradiomics (SUV_x_) were calculated separately and added to the RFs data set using Python packages Numpy (Version 1.18.1) and Simple ITK (Version 2.01). Full definitions of each radiomic feature are described in the Pyradiomics documentation. The SUV metrics are defined as follows:

- SUV 90^th^ Percentile—90% of the voxel’s SUV value fall below this number
- SUV mean—the mean SUV value in the region of interest
- SUV maximum—the maximum SUV value in the region of interest
- SUV *x* (*x* = 50, 60, 70, 80, 90)—mean of the voxels that are equal or greater than *x*% of SUV maximum

Extracted RFs and SUV metrics were harmonized using the ComBat method (neuroCombat, Version 0.2.7) (Online Resource 3). This method was first developed by Johnson et al^24^ for adjusting the batch effects in microarray data. Fortin et al adapted it for application to medical imaging^25^ and Orlhac et al applied it to PET radiomics.^26^ The effectiveness of ComBat was further verified by Da-Ano et al^27^ who also suggested improvements to the method. In this study the methods used by Fortin et al and Orlhac et al were used to reduce the effect of acquiring data with different scanners. The adjustments suggested by Da-Ano et al were not applied as they only gave small improvements and would be difficult to implement with the python library, neuroCombat, used. The dataset was grouped by scanner and each group was treated as a distinct batch. In retrospective studies these factors cannot be standardized without reducing the size of the dataset, so harmonization is recommended to minimize the effect. A list of all 107 RFs and SUV features used is provided in Online Resource 4. SUV metrics were used instead of target-to-blood pool ratio (TBR) as TBR is less frequently used within this clinical scenario, liver activity has become the common reference point.^10^

The effect of harmonization was evaluated with the Mann–Whitney *U* test. The null hypothesis that the two populations—the feature distribution for scanner *x* and *y*—were different populations (*P* < .05). Each pair of scanner groups were compared before and after harmonization for each of the 107 RFs and SUV metrics.

### Qualitative Grading of Vessel wall FDG Activity

A radiologist (co-author AFS) reanalyzed all scans and documented the vascular uptake score based on EANM/SNMMI guidelines^10^:

0. No uptake (mediastinum)
1. Low-grade uptake (*< *liver)
2. Intermediate-grade uptake (= liver), (possible aortitis)
3. High-grade uptake (*> *liver), (positive active aortitis)

### SUV Metrics and Radiomic Feature Diagnostic Utility Analysis

The diagnostic utility, also referred to as diagnostic performance, of a range of commonly used SUV metrics and extracted RFs was evaluated using two methods. Firstly, the Mann–Whitney *U* test was used. The *P* value for significance was adjusted using Bonferroni correction (> 0.05/number of features) to reduce the risk of false discovery related to multiple testing.

The second method of evaluating feature diagnostic utility was to use ML classifiers. The diagnostic utility of the ML classifiers was measured with area under the receiver operating characteristic curve (AUC) primarily, along with accuracy $\left(\frac{correct predictions}{all predictions}\right)$. As the literature value for AUC was 0.81 to 0.98^10^ any AUC value greater than 0.8 was considered a good performance. Logistic Regression (LR) classifiers were trained with SUV metrics and RFs individually (Sci-kit Learn Version 0.23.2). First the hyperparameters for each feature were tuned using the Sci-kit Learn function GridSearchCV where every combination of hyperparameters provided to the function was tested to find the optimal set.

Stratified five-fold cross-validation (CV) was used for both hyperparameter tuning and training of all final ML algorithms meaning the ratio of patients to controls in each fold was equal to the ratio in the total population. The AUC and the accuracy were both used to select the best performing hyperparameters. The tuned hyperparameters for each feature were used to train an LR model for that feature and the overall diagnostic utility was determined using the mean accuracy and mean AUC from stratified fivefold CV. Confidence Intervals (CI), in this case 95% CI, were determined using the standard error of the five testing AUCs and accuracies. Only training CV scores are reported in this study as splitting the data into training/test samples would be inappropriate for the sample size.^28^

### RF Signature Building

Many RFs can be extracted but not all of the derived features may provide useful information.^29^ Several RFs can be clustered together to achieve higher diagnostic performance than single features. However, using all available features retains a large amount of redundant information and creates noise in the final diagnostic model. Therefore, signatures of a smaller number of features were built to reduce the noise of the larger dataset while retaining the useful information provided. Three signatures were built using the methods described below.

### Performance Criteria and Correlation

The first method involved selecting features with high-individual diagnostic utility. For Signature A, features had to meet the following criteria: mean AUC ≥ 0.5, mean accuracy ≥ 0.7, and Mann-Whitney U test *p* value $\le$.05/*n*, where *n *= number of features (*n *= 107). The criteria was applied to the extracted feature results using Python package Pandas (Version 1.1.4) and the resulting features formed Signature A. Signature B was generated by removing highly correlated features from Signature A: for each pair of features, if the correlation coefficient was > 0.9, the feature with the lower AUC was removed.

### PCA

The number of features can be reduced using principal component analysis (PCA). PCA represents a large set of variables as a smaller set of principal components by finding relationships between features and combining them to reduce redundancy and minimize loss of information. PCA was applied using Sci-kit Learn (Version 0.23.2) and the number of PCs needed to account for 90% of the variance was retained. These PCs formed Signature C.

### Radiomic Signature Diagnostic Utility Analysis

Once the signatures were formed they were used as an input for a ML algorithm to diagnose active aortitis.^30–32^ To determine the best ML algorithm for distinguishing aortitis nine classifiers were built, trained and tested using Sci-kit Learn (Version 0.23.2): support vector machine, random forest, passive aggressive, LR, *k* nearest neighbors, perceptron, multi-layered perceptron, decision tree, and Gaussian process classification. The nine ML classifiers were trained on the radiomics signatures using the same methodology used for LR training on individual RFs (described in Section ‘SUV metrics and radiomic feature diagnostic utility analysis’). The determined hyperparameters for the three signatures are listed in Online Resource 5, 6, and 7. The best classifier for each signature was determined using the mean AUC of each classifier with a minimum mean accuracy of 80% or 70% if necessary.

## Results

### Patient Characteristics

Seventy-five participants were included, 50 of whom had a FDG PET–CT scan indicating active aortitis (Table 1). The age of the patients and female predominance reflects the typical demographic of patients with LVV, the commonest cause of which is GCA. The sensitivity of FDG PET–CT is significantly reduced within a few days of starting glucocorticoid treatment; doses were zero at the time of scanning unless stated otherwise.^33^ CRP (C-reactive protein) and ESR (Erythrocyte sedimentation rate) are biomarkers of systemic inflammation.

**Table 1:** Patient Demographics—at time of FDG PET–CT, clinical information within 4 weeks of FDG PET–CT

Characteristic	Aortitis	Controls
Participants	50	25
Age at time of scan, years—median (range)	60 (41–84)	68 (37–82)
Sex (male/female)	17/33	13/12
LVV type	GCA: 37, TAK: 4, IgG4 or RPF: 4, Misc: 5	n/a
Prednisolone dose (at time of scan, mg—median (range))	0 (0–40)*	0 (0–60)
Polymyalgic symptoms	yes (*n* = 15), no (*n* = 24), not known (*n* = 11)	n/a
Cranial symptoms	yes (*n* = 11), no (*n* = 25), not know (*n* = 14)	n/a
Claudication	yes (*n* = 12), no (*n* = 25), not known (*n* = 13)	n/a
CRP (mg/L) -median (range)	39 (5–164), not performed (*n* = 8), not known (*n* = 1)	n/a
ESR (mm/Hr) -median (range)	54 (0–143), not performed (*n* = 32), not known (*n* = 3)	n/a
Blood glucose (mmol/L) -median (range)	5.7 (4.2–9.9)	5.9 (4.2–12.0)

*12 Aortitis Patients were taking prednisolone at the time of scanning at the following doses: < 5 mg (*n* = 7), 20 mg (*n* = 1), 25 mg (*n* = 2), and 40 mg (*n* = 2)

*LVV* Large-Vessel Vasculitis, *GCA* Giant Cell Arteritis, *TAK* Takayasu’s arteritis, *IgG4* IgG4-related disease *RPF* Retroperitoneal Fibrosis, *n/a* Not Applicable, *CRP* C-reactive Protein, *ESR* Erythrocyte sedimentation rate

### Segmentation

The manual segmentation method was shown to be reproducible and accurate when compared to those performed by an experienced radiologist. Inter-observer variability scored an average Dice Coefficient of 0.91 (95% CI 0.90 to 0.92).

### Qualitative Grading

Guidelines, defined in Methods above, advocate qualitative grading of PET–CT scans based on FDG activity in the aortic wall relative to the liver^10^.Table 2 shows the grades assigned by an experienced radiologist on retrospective review of the images. Note the single aortitis patient who graded as 1 rather than 3 was taking 25 mg of prednisolone at the time reducing the sensitivity of FDG PET–CT.

**Table 2:** Grading of patient dataset based on the EANM/SNMMI guidelines^10^

Grade	No. of scans (aortitis)	No. of scans (control)	Ground truth diagnosis of aortitis	Ground truth diagnosis of no aortitis (control)
0	0	25	0	25
1	1	0	0	0
2	0	0	0	0
3	49	0	50	0

### Feature Harmonization

The Mann–Whitney *U* test was used to evaluate the effect of harmonization. The null hypothesis was defined as both feature distributions (before and after) being from the same population. The average *p* value increased in all cases as did the number of features where the null hypothesis was accepted (Table 3). When the two GE scanners were compared with the Mann–Whitney *U* test, we found sufficient difference that we chose to analyze them separately rather than combining the two into a single batch.

**Table 3:** Mann–Whitney *U* test results when feature distributions were compared before and after harmonization

	Before harmonization			After harmonization		
Scanners compared	1 vs 2	2 vs 3	1 vs 3	1 vs 2	2 vs 3	1 vs 3
Number of features where the null hypothesis was accepted (out of 107)	52	97	66	81	99	85
Average p value	0.148	0.224	0.144	0.199	0.230	0.182

*Scanner 1* GE Discovery 710, *Scanner 2* Phillips Gemini TF64, *Scanner 3* GE Discovery 690

### Diagnostic Utility of Harmonized SUV Metrics

**Figure 2:**
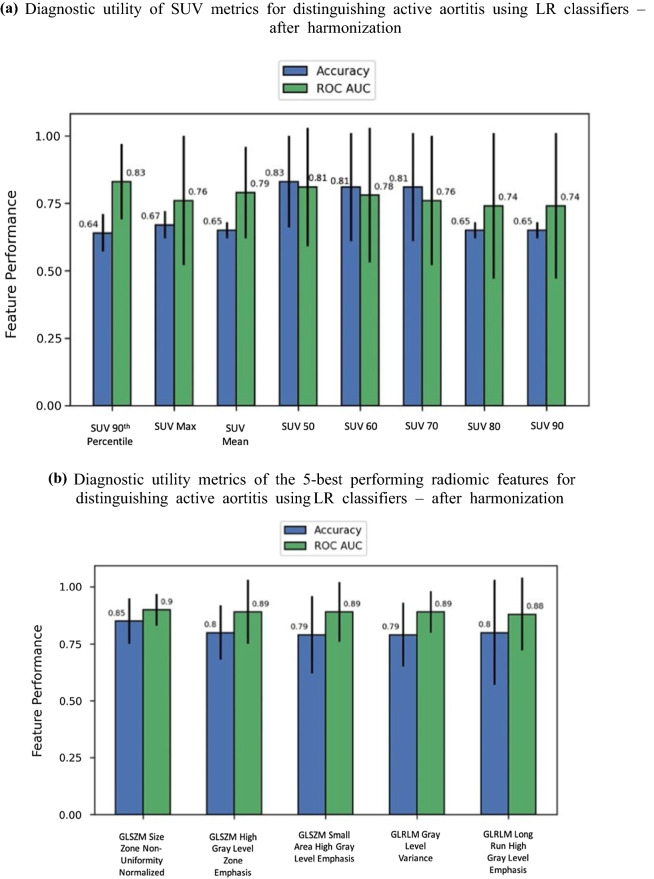

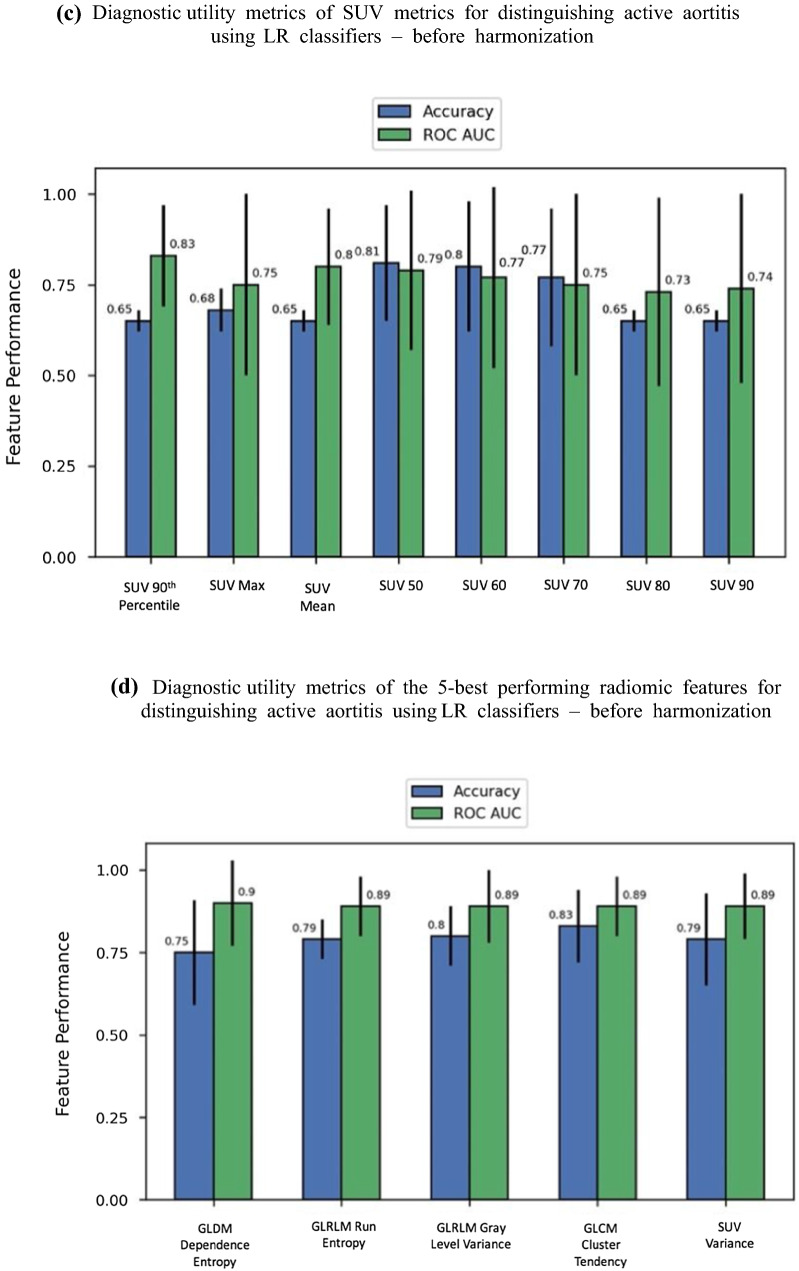
Diagnostic utility of SUV metrics and the 5-best performing radiomic features for distinguishing active aortitis. Before and after harmonization. *SUV* standardized uptake value, *GLDM* Gray-Level Dependence Matrix, *GLCM* Gray-Level Co-Occurrence Matrix, *GLRLM* Gray-Level Run Length Matrix, and *GLSZM* Gray-Level Size Zone Matrix. **a** Diagnostic utility of SUV metrics for distinguishing active aortitis using LR classifiers—after harmonization. **b** Diagnostic utility metrics of the 5-best performing radiomic features for distinguishing active aortitis using LR classifiers—after harmonization. **c** Diagnostic utility metrics of SUV metrics for distinguishing active aortitis using LR classifiers—before harmonization. **d** Diagnostic utility metrics of the 5-best performing radiomic features for distinguishing active aortitis using LR classifiers—before harmonization

All SUV metrics evaluated, except SUV_min_ and SUV 10th percentile, fulfilled the criteria based on the Mann–Whitney U test that there was a statistically significant difference between the mean metric value for the aortitis and control group (Bonferroni-corrected *P < *.00047). Figure 2a demonstrates the performance of harmonized SUV features in an LR classifier where higher accuracy and AUC indicate good diagnostic utility.

### Diagnostic Utility of Harmonized Radiomic Features

Using the Mann–Whitney *U* test 65/107 RFs demonstrated a statistically significant difference between the mean feature value for the aortitis and control group (Bonferroni-corrected *P < *.00047). The five-best performing RFs in terms of AUC, when used individually in an LR classifier, are shown in Figure 2b.

The performance of all SUV metrics and individual RFs in LR classifiers and in the Mann–Whitney U test can be viewed in Online Resource 8.

### Diagnostic Utility of Non-harmonized Features

Figure 2c and d shows the accuracy and AUC of non-harmonized SUV metrics and RFs, respectively. The 95% CI were too large to determine if there was a significant difference created by harmonization. The main difference between the two sets of results is a different set of RFs being ranked in the top five; however, overall performance was similar. No noticeable decrease in diagnostic utility, along with the results from the Mann–Whitney *U* test comparing scanner populations, justify retaining harmonization in the proposed methodology to improve generalizability.

### Correlation Between SUV Metrics and Best Performing Radiomic Features

Table 4 displays the correlation matrix of SUV metrics and the best performing RFs. It showed an intuitive split between the two groups but also emphasized that GLSZM Size Zone Non-Uniformity Normalized is only weakly correlated to other well-performing RFs. Table 5 displays the same information but for non-harmonized data.

**Table 4:**
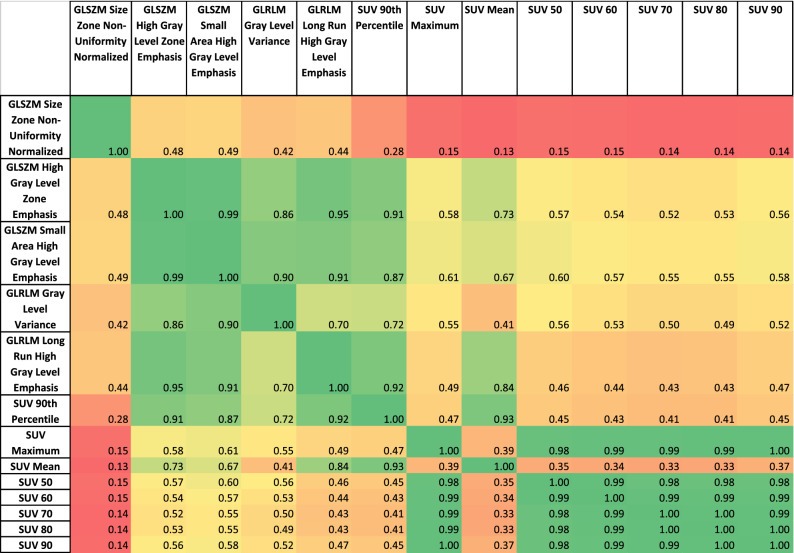
Correlation matrix of the best performing radiomic features and SUV metrics when harmonized

**Table 5:**
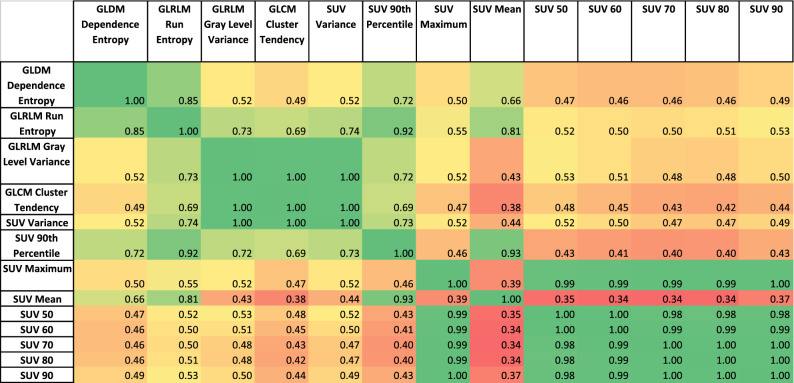
Correlation matrix of the best performing radiomic features and SUV metrics without harmonization

### Radiomic Feature Signature Building and Machine Learning

**Figure 3:**
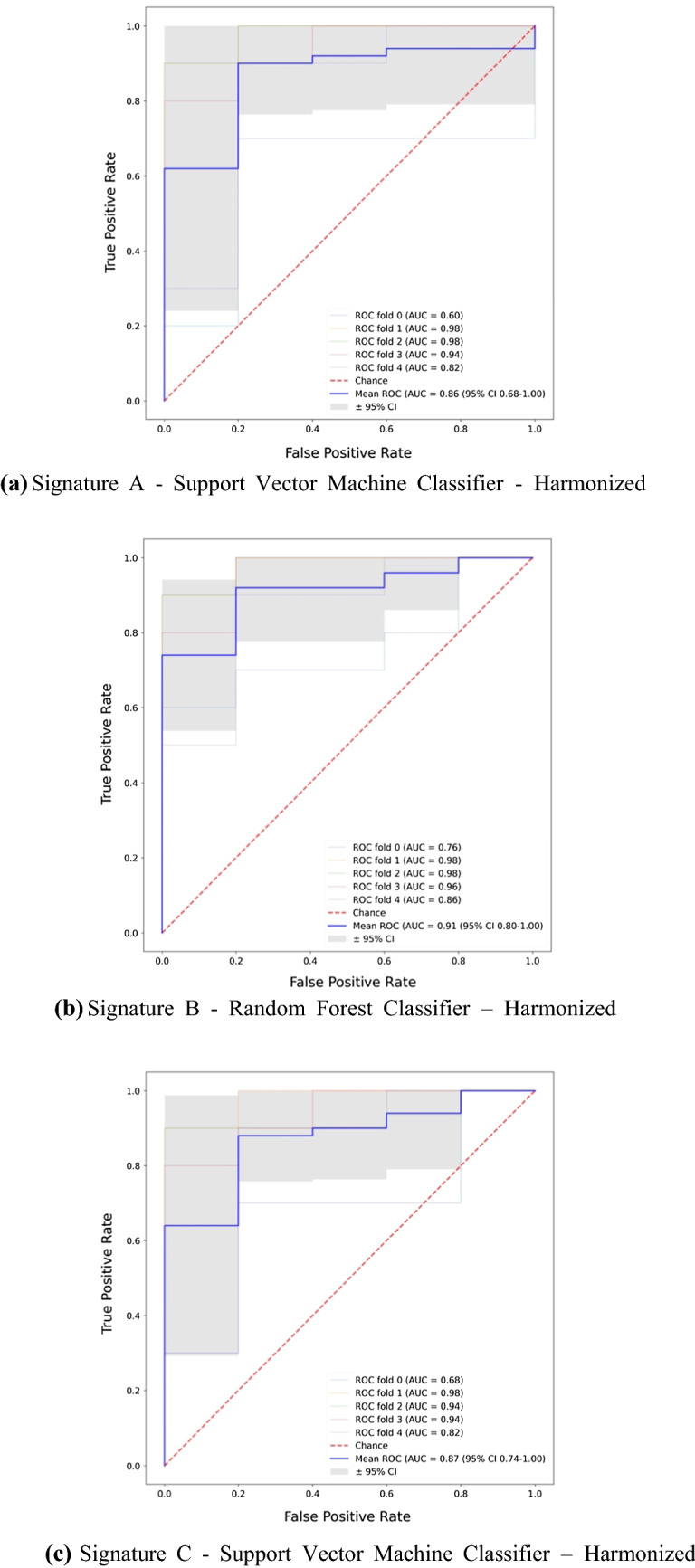

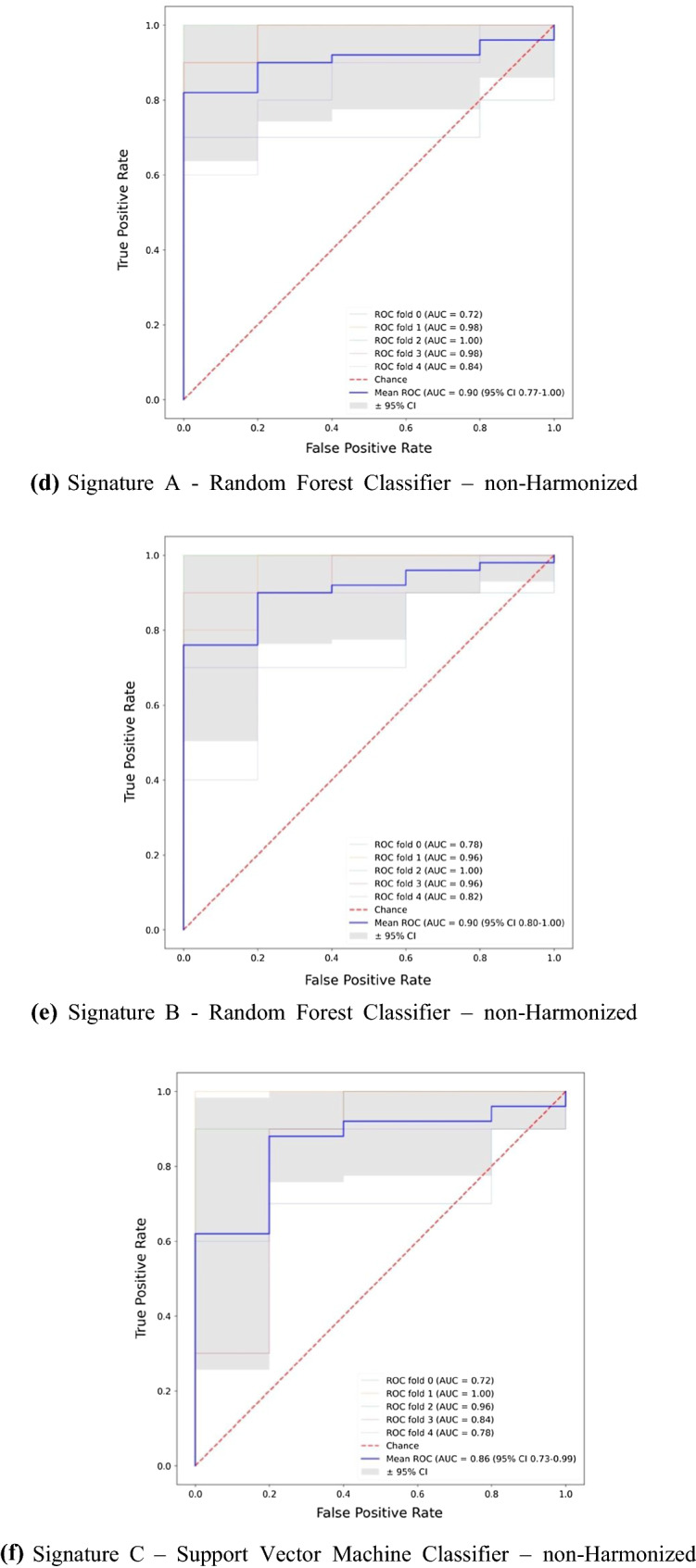
ROC curves of the best performing machine learning classifier trained on Signatures A, B, and C. **a** *Signature A* Support Vector Machine Classifier—Harmonized. **b ***Signature B* Random Forest Classifier—Harmonized. **c** *Signature C* Support Vector Machine Classifier—Harmonized. **d** *Signature A*—Random Forest Classifier—non-Harmonized. **e** *Signature B*—Random Forest Classifier—non-Harmonized. **f** *Signature C*—Support Vector Machine Classifier—non-harmonized

Signature A was based on passing minimum thresholds of diagnostic performance metrics. For this signature the best performing ML classifier was the support vector machine with an accuracy of 82.7% (95% CI 71.5 to 93.9%) and an AUC of 0.86 (95% CI 0.68 to 1.00). The ROC curve is shown in Figure 3a.

Signature B was built using the same thresholds but also removed highly correlated features. For this signature the best performing ML classifier was random forest with an accuracy of 84.0% (95% CI 72.8 to 95.2%) and an AUC of 0.91 (95% CI 0.80 to 1.00). The ROC curve is shown in Figure 3b. The results were not sensitive to the correlation threshold. Varying the threshold between 70 and 95% (generally considered range for high correlation) showed almost no variation in the best results. Some variations can be seen in the ML models that do not perform well, but these would not be utilized in a final analytical pipeline so were not considered important.

Online Resource 9 shows all accuracy (ACC_CV) and AUC (AUC_CV) results.

Six PCs were produced to account for 90% of the information in the original dataset. These PCs were used in Signature C. The best performing ML classifier was support vector machine with an accuracy of 82.7% (95% CI 71.5 to 93.9) and an AUC of 0.87 (95% CI 0.74 to 1.00). The ROC curve is shown in Figure 3c.

When the three signatures were built using non-harmonized features there was no significant change to results (Figure 3d–f). A slight improvement can be seen in Signature A when the data were not harmonized but overall, there is not enough evidence to select non-harmonized or harmonized as the superior method so both results were retained. The performance of all ML classifiers with Signatures A, B, and C can be viewed in Online Resources 10, 11, and 12, respectively.

### Summary of Diagnostic Performance

A summary of the diagnostic performance of each method is shown in Table 6. The AUC range presented for qualitative assessment were determined by a meta-analysis exploring the diagnostic accuracy of FDG PET–CT imaging in LVV.^10^ In the case of SUV metrics and RFs the best individual feature was determined by their AUC but with a minimum accuracy of 70%. The best SUV metric and radiomic feature for distinguishing aortitis was *SUV 90th percentile* and *GLSZM High Gray-Level Zone Emphasis*, respectively.

**Table 6:** Summary of the best diagnostic performance of each method

Method	AUC	AUC 95% CI	AUC	AUC 95% CI
Qualitative Assessment–Literature^10^	–	–	0.81–0.98	–
	Harmonized		Non-harmonized	
SUV Feature—SUV50%	0.81	0.22	0.81	0.14
Radiomic Feature—GLSZM Size Zone Non-Uniformity Normalized (harmonized)/GLDM Dependence Entropy(non-harmonized)	0.90	0.07	0.90	0.13
Signature A	0.86	0.18	0.90	0.13
Signature B	0.91	0.11	0.90	0.11
Signature C	0.87	0.14	0.86	0.14

*SUV* standardized uptake value, *GLDM* Gray-Level Dependence Matrix, *GLCM* Gray-Level Co-Occurrence Matrix, *GLRLM* Gray-Level Run Length Matrix, *GLSZM* Gray-Level Size Zone Matrix

## Discussion

The purpose of this study was to develop a methodological framework to support AI-assisted diagnosis of active aortitis, using ML classifiers trained with RFs from FDG PET-CT. The best performing individual RF had an AUC of 0.9 (95% CI 0.83 to 0.97) when harmonized (*GLSZM Size Zone Non-Uniformity Normalized*), similar to the current clinical standard of qualitative assessment (AUC = 0.81-0.98^10^). The three signatures performed similarly to the best performing individual RFs. Signature B has the highest mean AUC of any of the proposed methods with an AUC of 0.91 (95% CI 0.80 to 1.00). There was no clear consensus on whether harmonization improved diagnostic performance. In most cases a similar result was achieved using harmonization with the exception of Signature A. Therefore, both methodologies will be retained for future validation. This method has potential to be used as an automated quantitative analysis tool alongside standard clinical assessment toward a more rapid, objective, and standardized evaluation of aortitis.

Visual scores were assigned as part of this study using the EANM/SNMMI grading guidelines^10^ following the results of Stellingwerf et al^34^ who demonstrated a high diagnostic accuracy when arterial FDG uptake was compared to liver FDG uptake. The visual scores demonstrated good agreement with ground truth diagnoses (Table 2) and are easy to perform, but subjective. Subjective assessment risks inter-observer variability. Qualitative assessment has been reported to have good inter-observer agreement when the score comprises a limited number of categories^35^ but this is less well established in aortitis than in other areas, such as lymphoma response assessment. No published data were found on the effect of observer experience on visual assessment in this clinical scenario. As borderline cases were not used in the analysis, all but one case was graded as either 0 or 3 meaning there was no uptake or high-grade uptake, respectively. One case was graded as 1 (low-grade uptake); this reduced signal was a result of prednisolone treatment (25 mg daily) which diminishes PET sensitivity.^33^ A similar scoring system based on arterial uptake across different regions was proposed by Grayson et al named PET Vascular Activity Score (PETVAS).^13^ PETVAS is not routinely used in clinical practice as it is time consuming. Kang et al showed that PETVAS is superior to SUV_max_, but it is unclear if it is better than a single visual score assigned using the EANM/SNMMI guidelines.^36^ SUV metrics were used instead of target-to-blood pool ratio (TBR) as TBR is less frequently used in aortitis where liver activity has become the common reference point.^10^

The diagnostic utility of semi-quantitative measurements using SUV, which are widely utilized in PET, was compared against other features for detecting active aortitis. In LR classifiers, SUV metrics with high mean AUC values had a broad 95% CI range making them less useful candidates when compared to qualitative assessment. Overall SUV metrics had some diagnostic utility in Mann–Whitney U and LR classifier testing.

The performance of *SUV*_*max*_ is affected by noise.^37^ Similarly, atherosclerosis can be associated with FDG activity and although patients and controls with a large amount of atherosclerotic plaque were removed from the cohort, some degree of the condition is present in the relevant age group.^38^ Together, these two factors may have lowered the diagnostic utility of *SUV*_max_. The ability to reliably distinguish aortitis from atherosclerosis will need to be considered in any automated diagnostic methods. *SUV*_*x*_ also relies on *SUV*_max_. In particular, *SUV*_50_ performs better than other *SUV*_*x*_ metric, probably because it covers a larger percentage of the voxels, so the effect of noise and bright patches is mitigated. *SUV*_mean_ and *SUV*_50_ would likely perform better if only active tissue had been included in the ROI rather than the whole aorta.

RFs demonstrating the highest mean diagnostic utility focus mainly on high gray levels and heterogeneity. The *GLSZM Size Zone Non-Uniformity Normalized* was the best RF according to AUC and performed well in terms of accuracy and the Mann–Whitney U test. Its value is higher in active aortitis than controls, which means there is more heterogeneity in zone size volumes in aortic imaging. This is an expected finding and reflects greater metabolic activity in the aortic wall of patients with active aortitis than in controls. The importance of high gray values and zones and heterogeneity is further emphasized in other RFs with high diagnostic utility. The addition of heterogeneity to quantitative diagnostic models in aortitis may help improve performance.

## Limitations

Limitations of the study include the retrospective single-center design, relatively small cohort, imbalanced dataset, lack of an automated segmentation, lack of independent testing, and need for external validation of initial findings.^39^ The cohort size is an important consideration when designing a radiomic study. Small cohort sizes relative to the number of RFs can introduce overfitting and type 1 errors.^40,41^ Bonferroni correction and feature reduction were used to reduce these issues but overfitting is still plausible. Sollini et al concluded in their systematic review that the lack of external validation was the key issue preventing radiomics translating into routine clinical practice.^42^

As this study used PET images from multiple scanners the images had to be resampled to attain a uniform voxel size across the entire data set. Therefore, the images were downsampled to 4 mm^3^ which results in loss of resolution that can be considered a limitation. However, there is no consensus on whether downsampling or upsampling should be chosen in this situation^43^; it is arguably a more cautious approach to reduce than create data. Downsampled data are less computationally expensive to analyze allowing easier transferability and making the process more scalable for clinical applications. Downsampling to 4 mm^3^ also had the advantage of being an integer size allowing for more precision.

Finally, another important limitation is that AUCs are difficult to compare. Delong’s method^44^, which is commonly used for this practice, is regarded as a controversial method for AUC comparison and there is no other well-accepted scientific approach to properly compare AUCs. Delmier et al state that two models developed and tested on the same data should not be compared with Delong’s method as it would lead to a low powered test with a conservative result.^45^ Thus, in this study any conclusions drawn concerning AUCs need to be considered with caution.

### New Knowledge Gained

The initial analysis established that a method using radiomics and ML classifiers has the potential to assist in the diagnosis of active aortitis. Previously the utility of radiomics in aortitis had not been established with the most similar work being performed using SUV metrics alone. In harmonized data, the SUV metric with the highest AUC score, while also having an accuracy above 70%, was SUV_50_ with an AUC of 0.81 (95% CI 0.59 to 1.00). The RF that met these criteria was GLSZM Size Zone Non-Uniformity Normalized with AUC = 0.90 (95% CI 0.83 to 0.97). When signatures were formed with groups of RFs the highest AUC was scored by Signature B, using high performing features that were not highly correlated, with AUC = 0.91 (95% CI 0.80 to 1.00).

### Future Work

In the future, it is envisaged that this method has the potential to be automated, fast, and standardize PET–CT imaging-based diagnosis of aortitis, reducing human error and opening up possibilities for more precise quantification of inflammation burden for disease monitoring and prognosis. The methodology proposed here could be implemented in clinical practice to aid diagnosis, reducing variation between observers and improving diagnostic accuracy of aortitis in patients who have already started treatment or who have co-incidental atherosclerosis.^46^ This includes external validation of the methodology using multi-center datasets.^47^

TRIPOD guidelines were followed to assure the completeness of our method.^18,48^ Other scores such as the Radiomics Quality Score (RQS) were referred to but contained aspects beyond the scope of the methodology proposed in this study.^49^ RQS and the aspects it discusses such as external validation will be implemented more thoroughly in future work. Other work leading on from this study includes automating segmentation.^50–52^ Doing so would increase the efficiency of the analysis pipeline and improve reproducibility,^53^ which is important as several studies have reported that RFs can be sensitive to the segmentation method^54–57^

Following on from this diagnostic method, potential future work could include more specific classification similar to the visual grading (defined in the Methods).^10^ Analysis could also go further and predict outcome and treatment response once a larger cohort is available.

## Conclusion

The purpose of this study was to develop a methodological framework for assisted diagnosis of active aortic inflammation using RF and SUV metrics derived from FDG PET–CT. Selected RFs and SUV metrics had high accuracy and AUC scores when used individually in LR classifiers. ML classifiers trained on radiomic signatures had similar diagnostic performance to individual RFs. This demonstrates that a radiomic method for assisted diagnosis of active aortitis may be proven feasible, pending further validation, eventually opening up the potential for automated and standardized diagnosis of aortitis.

## Supplementary Information

Below is the link to the electronic supplementary material.

Supplementary file1 (PPTX 525 kb)

Supplementary file2 (MP3 2579 kb)

## Abbreviations

RF: Radiomic feature
SUV: Standardized uptake value
PCA: Principal component analysis
LR: Logistic regression
LVV: Large-vessel vasculitis
FDG PET–CT: [^18^F]-Fluorodeoxyglucose Positron Emission Tomography–Computed Tomography
GCA: Giant cell arteritis
