# Supplementary material for: A methodological framework for AI-assisted diagnosis of active aortitis using radiomic analysis of FDG PET–CT images: Initial analysis
Source: J Nucl Cardiol. 2022 Mar 23;29(6):3315–31. doi: 10.1007/s12350-022-02927-4 (PMC9834376; doi:10.1007/s12350-022-02927-4)
Supplement: Supplementary file 1 — Supplementary file1 (PPTX 525 kb) [file 12350_2022_2927_MOESM1_ESM.pptx]

## Slide 1
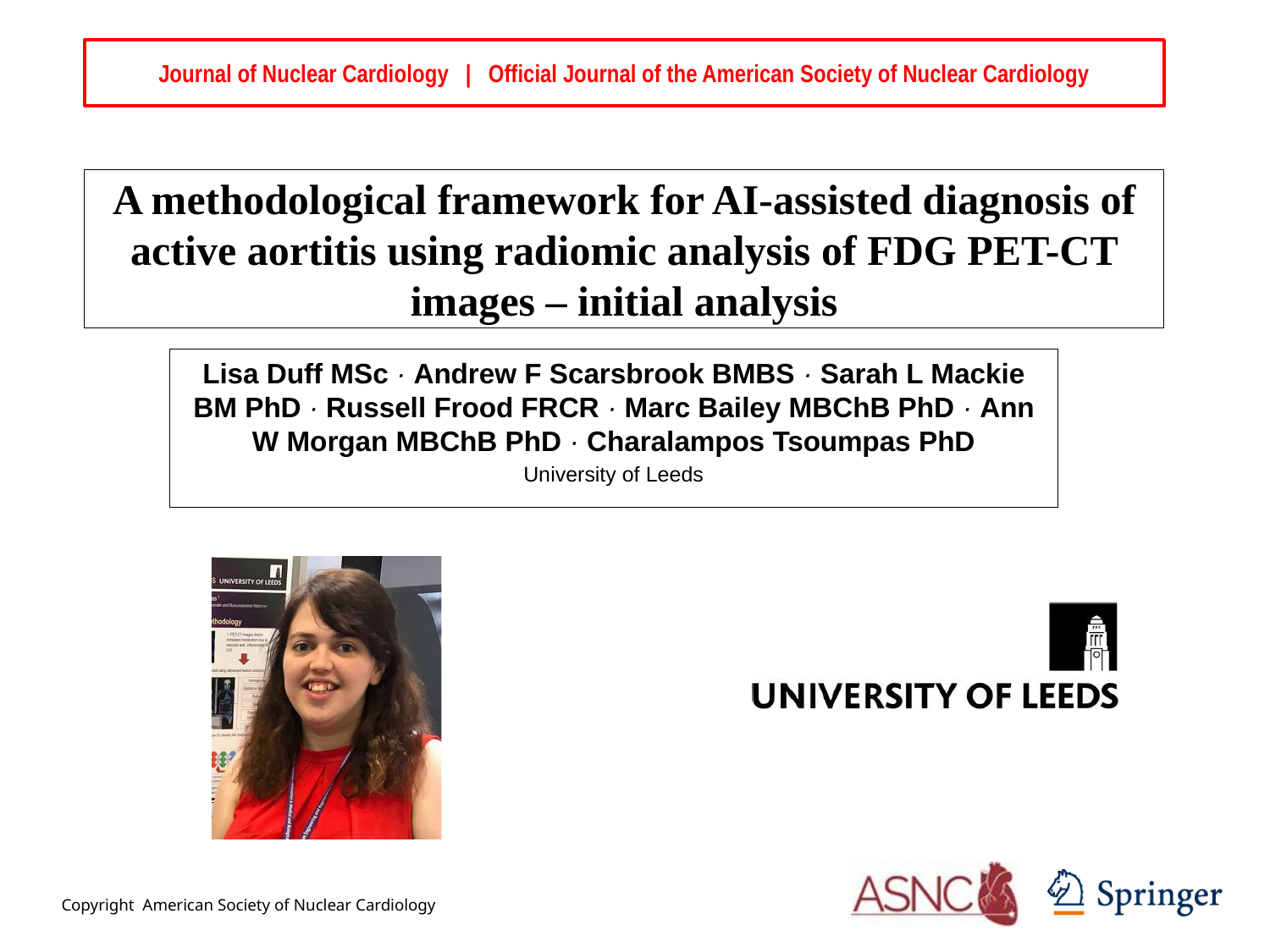

Journal of Nuclear Cardiology | Official Journal of the American Society of Nuclear Cardiology
# A methodological framework for AI-assisted diagnosis of active aortitis using radiomic analysis of FDG PET-CT images – initial analysis
Lisa Duff MSc · Andrew F Scarsbrook BMBS · Sarah L Mackie BM PhD · Russell Frood FRCR · Marc Bailey MBChB PhD · Ann W Morgan MBChB PhD · Charalampos Tsoumpas PhD
University of Leeds
Copyright American Society of Nuclear Cardiology

## Slide 2
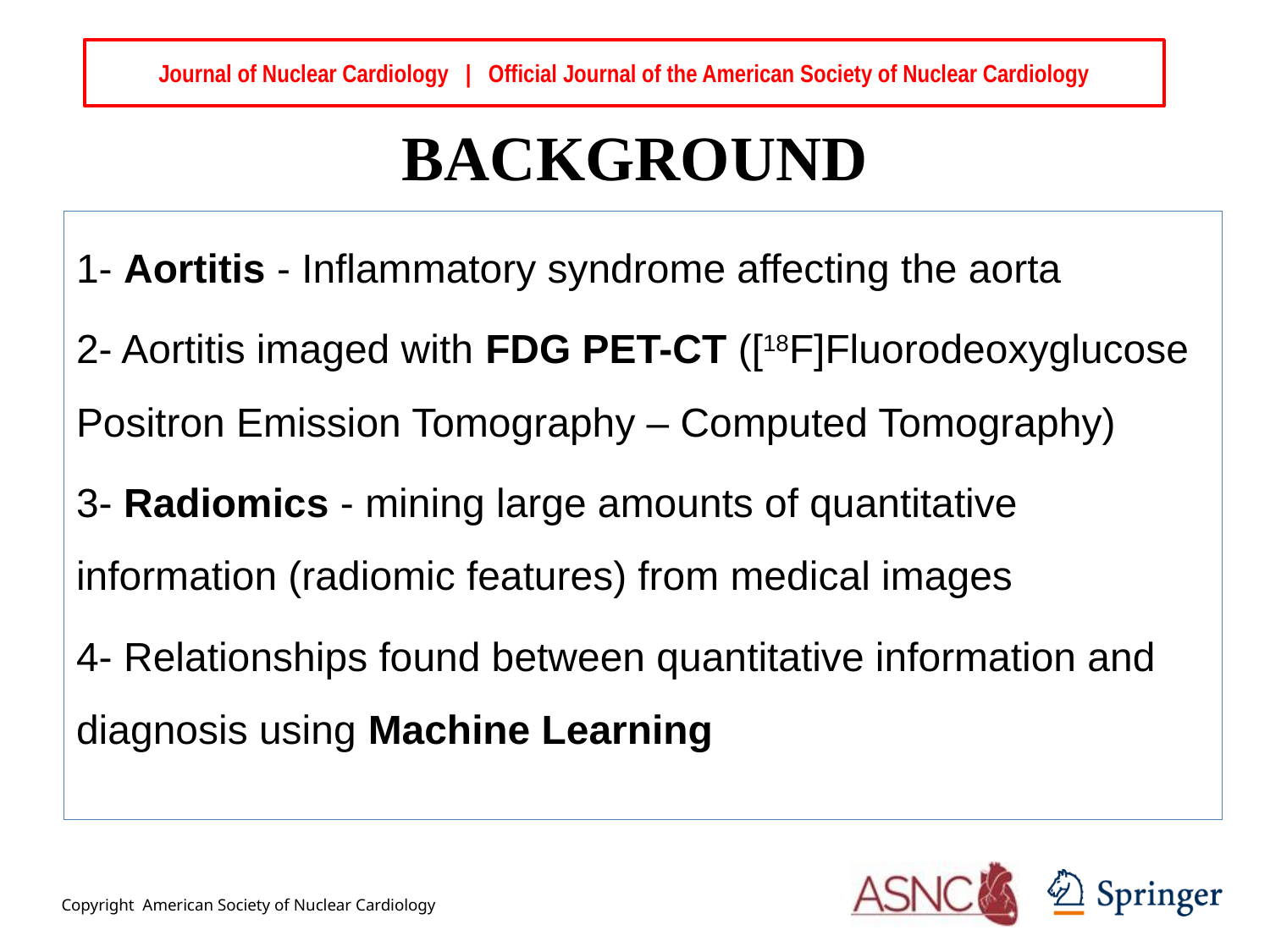

Journal of Nuclear Cardiology | Official Journal of the American Society of Nuclear Cardiology
# BACKGROUND
1- Aortitis - Inflammatory syndrome affecting the aorta
2- Aortitis imaged with FDG PET-CT ([18F]Fluorodeoxyglucose Positron Emission Tomography – Computed Tomography)
3- Radiomics - mining large amounts of quantitative information (radiomic features) from medical images
4- Relationships found between quantitative information and diagnosis using Machine Learning
Copyright American Society of Nuclear Cardiology

## Slide 3
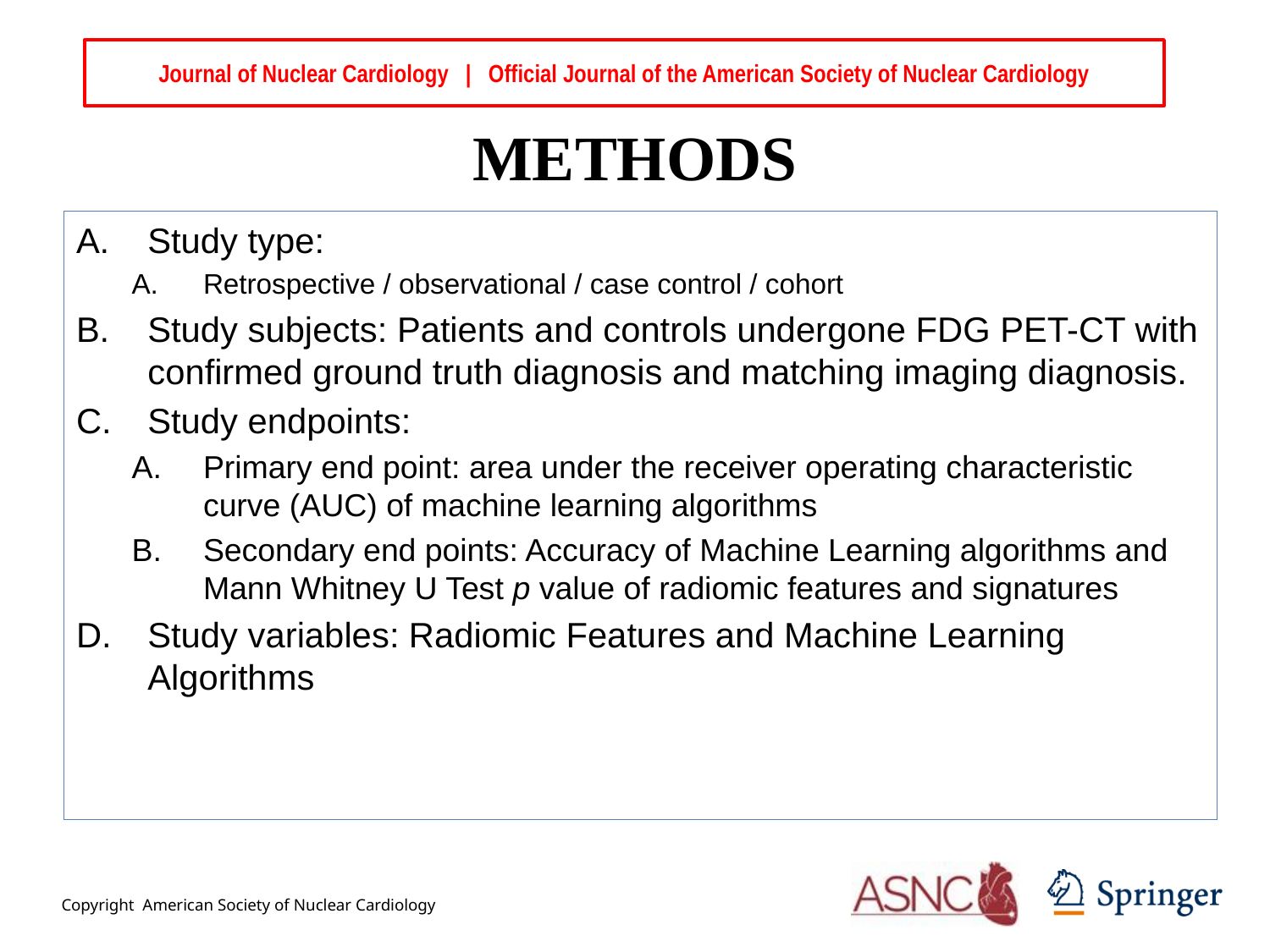

Journal of Nuclear Cardiology | Official Journal of the American Society of Nuclear Cardiology
# METHODS
Study type:
Retrospective / observational / case control / cohort
Study subjects: Patients and controls undergone FDG PET-CT with confirmed ground truth diagnosis and matching imaging diagnosis.
Study endpoints:
Primary end point: area under the receiver operating characteristic curve (AUC) of machine learning algorithms
Secondary end points: Accuracy of Machine Learning algorithms and Mann Whitney U Test p value of radiomic features and signatures
Study variables: Radiomic Features and Machine Learning Algorithms
Copyright American Society of Nuclear Cardiology

## Slide 4
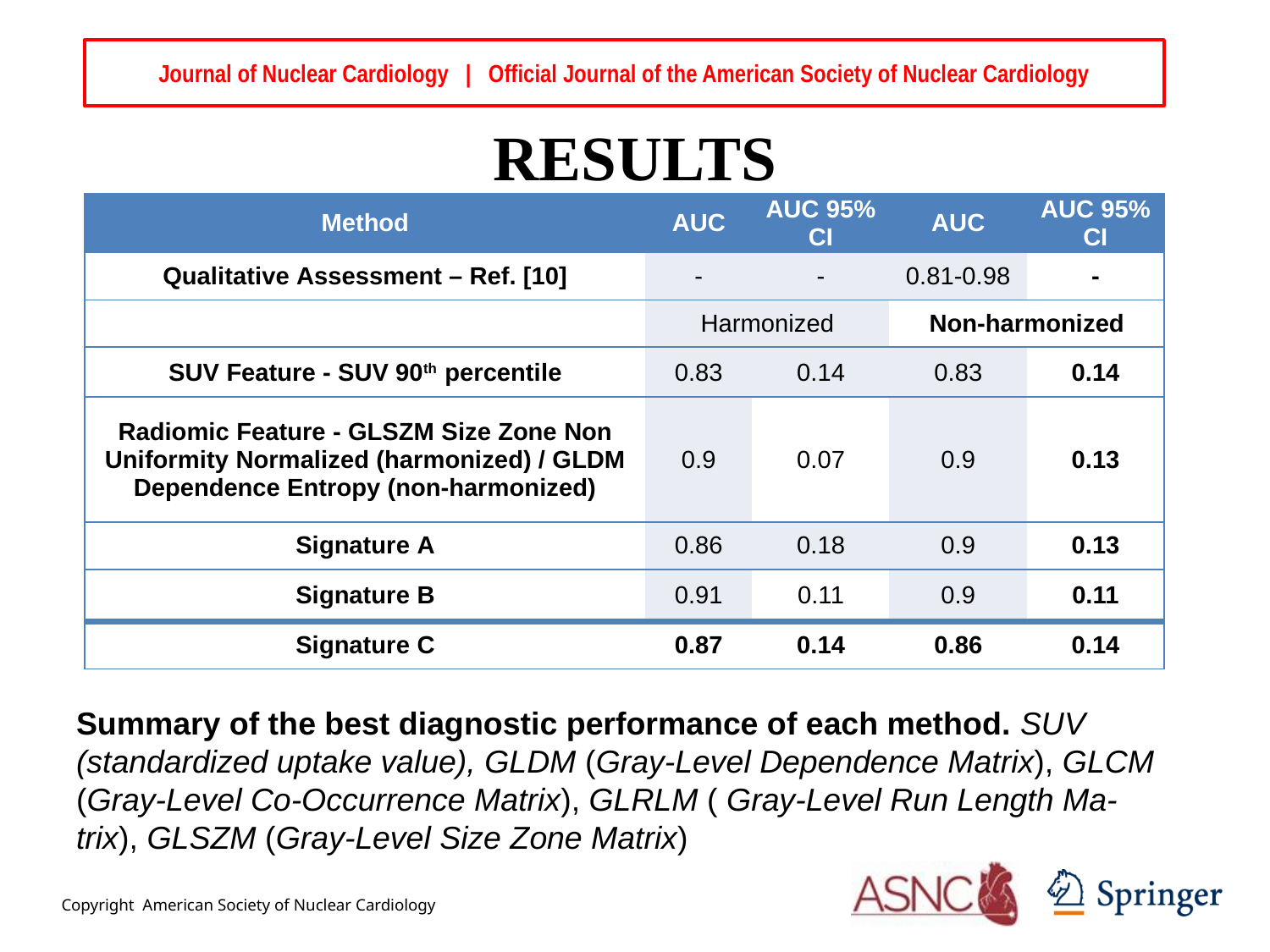

Journal of Nuclear Cardiology | Official Journal of the American Society of Nuclear Cardiology
# RESULTS
| Method | AUC | AUC 95% CI | AUC | AUC 95% CI |
| --- | --- | --- | --- | --- |
| Qualitative Assessment – Ref. [10] | - | - | 0.81-0.98 | - |
| | Harmonized | | Non-harmonized | |
| SUV Feature - SUV 90th percentile | 0.83 | 0.14 | 0.83 | 0.14 |
| Radiomic Feature - GLSZM Size Zone Non Uniformity Normalized (harmonized) / GLDM Dependence Entropy (non-harmonized) | 0.9 | 0.07 | 0.9 | 0.13 |
| Signature A | 0.86 | 0.18 | 0.9 | 0.13 |
| Signature B | 0.91 | 0.11 | 0.9 | 0.11 |
| Signature C | 0.87 | 0.14 | 0.86 | 0.14 |
Summary of the best diagnostic performance of each method. SUV (standardized uptake value), GLDM (Gray-Level Dependence Matrix), GLCM (Gray-Level Co-Occurrence Matrix), GLRLM ( Gray-Level Run Length Ma- trix), GLSZM (Gray-Level Size Zone Matrix)
Copyright American Society of Nuclear Cardiology

## Slide 5
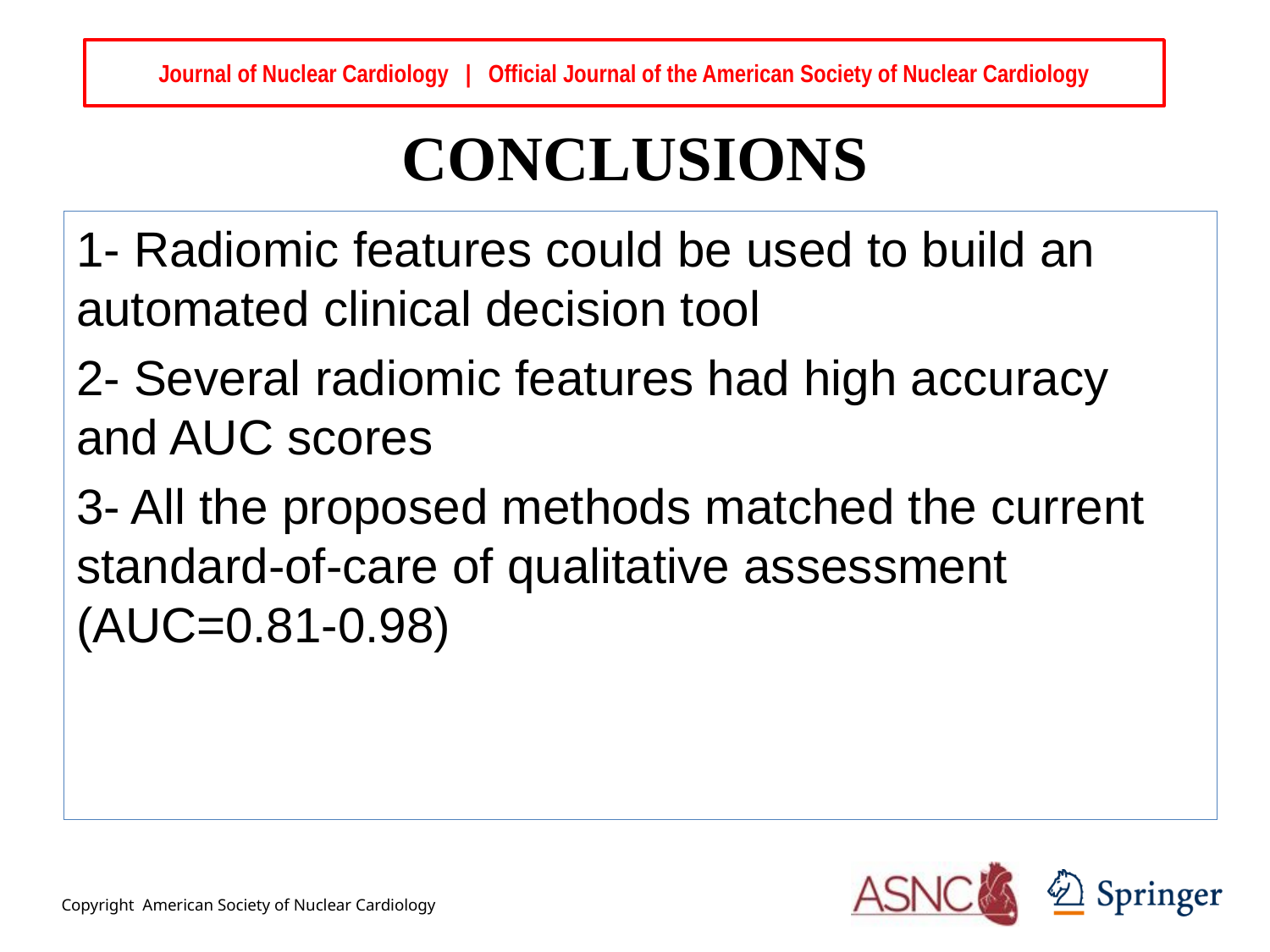

Journal of Nuclear Cardiology | Official Journal of the American Society of Nuclear Cardiology
# CONCLUSIONS
1- Radiomic features could be used to build an automated clinical decision tool
2- Several radiomic features had high accuracy and AUC scores
3- All the proposed methods matched the current standard-of-care of qualitative assessment (AUC=0.81-0.98)
Copyright American Society of Nuclear Cardiology
